# Acupuncture Improved the Function of the Lower Esophageal Sphincter and Esophageal Motility in Chinese Patients with Refractory Gastroesophageal Reflux Disease Symptoms: A Randomized Trial

**DOI:** 10.1155/2023/4645715

**Published:** 2023-05-24

**Authors:** Tang Yuming, Zhao Yuping, Lin Yihan, Zhu Ying, Huang Jia, Shen Hanbing, Zou Duowu, Yao Weiyan

**Affiliations:** ^1^Department of Gastroenterology, Shanghai Jiao Tong University Medical School Affiliated Ruijin Hospital, Shanghai, China; ^2^Department of Gastroenterology, Chengdu First People's Hospital, Chengdu, Sichuan, China; ^3^Department of Gastroenterology, Fujian Provincial Hospital, Fuzhou, Fujian, China; ^4^Department of Acupuncture and Moxibustion, Long Hua Hospital, Shanghai, China

## Abstract

**Objectives:**

Acupuncture is therapeutic for refractory gastroesophageal reflux disease by an unclear mechanism. This study was aimed at investigating the effect of acupuncture on esophageal motility in patients with symptoms of refractory gastroesophageal reflux disease.

**Methods:**

Sixty-eight patients with refractory gastroesophageal reflux disease symptoms were prospectively enrolled from August 2014 to December 2018 and randomized into acupuncture and control groups (*n* = 33 and 35, respectively). The acupuncture group received acupuncture, and the control group received sham acupuncture. Pre- and post-acupuncture high-resolution manometry was performed to evaluate the effect of acupuncture on esophageal motility. The GerdQ questionnaire was used to evaluate the pre- and post-intervention symptoms.

**Results:**

After acupuncture, there was a significant increase in the length of lower esophageal sphincter (3.10 ± 1.08 cm vs. 3.78 ± 1.01 cm), length of intra-abdominal lower esophageal sphincter (2.14 ± 1.05 cm vs. 2.75 ± 1.16 cm), and mean basal pressure of lower esophageal sphincter (22.02 ± 10.03 mmHg vs. 25.06 ± 11.48 mmHg) in the acupuncture group (*P* = 0.014); moreover, the numbers of fragmented contraction and ineffective contraction decreased from 36 to 12 (*P* < 0.001) and 43 to 18 (*P* = 0.001), respectively, in the acupuncture group. However, no significant difference was observed in the control group. The GerdQ score decreased significantly from 9.45 ± 2.44 to 7.82 ± 2.21 points in the first week after acupuncture (*P* < 0.001).

**Conclusions:**

Acupuncture, which improves esophageal motility, has short-term efficacy in patients with symptoms of refractory gastroesophageal reflux disease. This trial is registered with Chinese Clinical Trial Registry (ChiCTR1800019646).

## 1. Introduction

Gastroesophageal reflux disease (GERD) refers to a series of uncomfortable symptoms and complications including gastrointestinal bleeding, esophageal stricture, asthma, and reflux laryngitis [1, 2] caused by gastric content reflux. Proton pump inhibitors (PPIs) are the standard therapy for GERD. Endoscopic finding of esophagitis or pathological reflux detected by 24-hour esophageal pH monitoring has been regarded as the gold standard for GERD. However, approximately 40% of patients do not experience symptom relief with the standard treatment course of 4 ~ 8 weeks, and their condition is referred to as refractory gastroesophageal reflux disease (rGERD) [3]. The pathophysiological mechanism of rGERD is multivariate, including an insufficient PPI dose, improper medication time, acid breakthrough at night, *CYP2C19* polymorphism, high esophageal sensitivity, and non-acid reflux [4]. Gastroesophageal dysmotility plays an important role in GERD pathogenesis, principally because of the defects of the anti-reflux mechanism and decreased esophageal clearance [5].

Many studies have shown that acupuncture can improve GERD symptoms [6]. Recent studies showed acupuncture with a double PPI dose resulted in better regurgitation control [7, 8]. Kappelle et al. confirmed that lower esophageal sphincter electrical stimulation therapy (LES-EST) significantly improved reflux symptoms and esophageal acid exposure in GERD patients with unsatisfactory PPI efficacy [9]. However, few data were focused on the effect of acupuncture on esophageal motility. Hence, we aimed to evaluate esophageal motility changes by using high-resolution manometry (HRM) and corresponding symptom changes after acupuncture therapy in patients with rGERD symptoms.

## 2. Patients and Methods

### 2.1. Study Design

**Figure 1:**
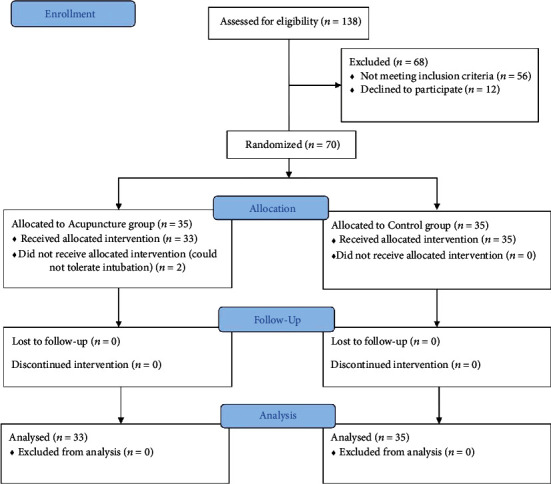
Flow chart of the study in refractory gastroesophageal reflux disease.

This was a single-center, randomized, parallel, controlled trial conducted from August 2014 to December 2018.  Figure 1 shows the study flow chart. Participants were randomized to acupuncture and control groups in a 1 : 1 ratio. The SPSS 24.0 software was used to generate random numbers for the study. The sample size was estimated according to the difference in the effective rate in the acupuncture group (two-sided test): *N* = {*Zα*/2[(2*P*_mean_)(1 − *P*_mean_)(*Q*1 − 1 + *Q*2 − 1)]0.5 + *Zβ* [*P*1*Q*1 − 1(1 − *P*1) + *P*2*Q*2 − 1(1 − *P*2)]0.5}^2^/(*P*1 − *P*2)^2^, where *Q* was each group's sample proportion, *P* was each group's effective treatment rate, and *N* was the total number of cases. Based on previous studies, the probability of the acupuncture and control group was 40% and 0%, respectively, allowing for 5% type I errors (*α* = 0.05, one-sided); the test power was 90% (*β* = 0.1). The required sample size of *n* = 58.2 was obtained. Considering a loss of 20% of the participants, enrollment of 35 participants was planned for each of the groups. The efficacy assessor, operator, and statistician were separated.

### 2.2. Patients

The inclusion criteria were as follows: age of 18–70 years; typical reflux-related symptoms such as acid reflux and heartburn, with or without some atypical symptoms such as chest pain, retrosternal discomfort, and pharyngeal discomfort; reflux esophagitis or Barrett's esophagus confirmed by gastroscopy or abnormal acid reflux confirmed by esophageal 24-hour pH monitoring off PPI; and nonresponse to standard PPI doses for at least an 8-week continuous treatment (oral PPIs including rabeprazole, omeprazole, esomeprazole, lansoprazole, and pantoprazole). For a diagnosis of Barrett's esophagus [10], it was necessary that endoscopy showed columnar mucosa extending above the gastroesophageal junction, lining the distal esophagus, and detection of intestinal metaplasia with goblet cells in esophageal biopsy.

The exclusion criteria were as follows: peptic ulcer, Zollinger–Ellison syndrome, primary esophageal motility disorders (e.g., achalasia), primary esophageal fistula, upper gastrointestinal malignancy, drug-induced esophagitis, nasopharyngeal or esophageal obstruction, and a history of gastroesophageal or duodenal surgery; severe mental illness or cognitive disorder, which may not allow patient co-operation; severe heart/lung/cerebrovascular disease, hematopoietic diseases, or coagulation dysfunction; pregnancy and lactation; skin lesions and skin infections; and other acupuncture contradictions.

### 2.3. Protocol for Esophageal HRM

The patients were asked to fast for at least 8 hours to reduce emesis and aspiration during intubation and discontinue drugs that may affect esophageal motility for 48 hours (including calcium channel blockers, nitrates, prokinetics, loperamide, adrenergic antagonists, opiate antagonists or agonists, anticholinergic agents, tricyclic antidepressants, caffeine, and nicotine). The Manoscan 360 system (Given Scientific Instruments Inc, Israel) was used. It consists of a solid-state manometric catheter connected to 36 circumferential pressure sensors spaced at 1-cm intervals and ManoView acquisition software, which allows real-time display of pressure data for esophageal body and sphincters. After automated calibration, the manometric catheter was extended to the stomach through a nostril with the patient upright and then in the supine position to collect data. After the adaptation period, resting-state data of the esophagus were recorded for 30 seconds to determine the location and resting pressure of the upper and lower sphincters. Furthermore, the patients were asked to drink 5 mL of water 10 times, each at 20-second intervals, to assess LES relaxation and esophageal body and upper esophageal sphincter functions. All participants underwent pre- and post-intervention HRM to evaluate the acute effect of acupuncture, without the manometric catheter removed until the end of the whole procedure.

### 2.4. Acupuncture Procedure

Acupuncture was performed by the same registered acupuncturist. After the participants' first HRM examination in the supine position, patients in the acupuncture group received acupuncture for 30 minutes at the bilateral Zusanli (ST36), Neiguan (PC6), and Gongsun (SP4) acupoints. Disposable needles (0.25 × 40 mm; Huatuo) were vertically inserted approximately 30 mm in depth at these acupoints, and then, twirling, lifting, and thrusting (once every 5 minutes) manipulations were performed to reach acupuncture de qi (soreness, heaviness, and distension sensation when needling). The control group received shallow needling at bilateral sham ST36, PC6, and SP4 (nonacupoints located at physical locations different from ST36, PC6, and SP4) (Supplement, available at http://www.annals.org). Needles were inserted vertically 3–5 mm into the nonacupoints without manipulation. After the intervention, the subjects were observed for 1 hour; any adverse events were preliminarily determined by the doctor. If necessary, patients would be further examined or hospitalized. For typical symptoms, GerdQ scores were recorded at baseline and followed up each week for a total of 1 month to evaluate the long-term effect of acupuncture. Regarding atypical symptoms, the descriptors “mild,” “moderate,” and “severe” were used for severity and “improved,” “unchanged,” and “aggravated” for treatment response.

### 2.5. Data Analysis

Manoview Analysis version 3.0 software (Given Scientificu Instruments Inc, Israel) was used for data analysis. The primary outcomes were normal contraction (distal contractile integral [DCI]: >450 mmHg s cm but <8000 mmHg s cm) and intact contraction (without a large break [>5 cm length] in the 20-mmHg isobaric contour and with DCI of >450 mmHg s cm and distal latency [DL] ≥ 4.5 s), while the secondary outcomes were the length of LES, LES pressure (LESP), integrated relaxation pressure (IRP), upper esophageal sphincter pressure (UESP), and UES relaxation pressure. The normal ranges were as follows: LES length: 2.7–4.8 cm; LES basal pressure: 13–43 mmHg; UES basal pressure: 34–104 mmHg; UES relaxation pressure: <12 mmHg; DCI: 450–8000 mmHg s cm; IRP: ≤15 mmHg; and DL: ≥4.5 s. Esophageal HRM was interpreted according to the Chicago classification version 3.0. Recording for 24-hour esophageal pH monitoring was performed with a multi-use VersaFlex® catheter (Given Scientific Instruments Inc, Los Angeles, CA, USA). All the patients' data were recorded on Digitrapper® equipment (Given Scientific Instruments Inc, Los Angeles, CA, USA). Abnormal esophageal acid exposure was defined as total percent time of pH < 4 greater than 4% and DeMeester score >14.7 [11].

### 2.6. Statistical Analysis

Statistical analysis was performed using SPSS 24.0 software. The measurement data of normal distribution were expressed as $\bar{x}\pm s$. The measurement data of non-normal distribution were represented by median (quartile). The counting data were expressed as number of cases and percentages. Pre- and post-treatment outcome variables were analyzed, and according to data characteristics, *t*-test, chi-square test, or rank-sum test was chosen. Spearman's correlation coefficient was used for the correlation analysis. A statistically significant difference was defined as *P* < 0.05.

## 3. Results

### 3.1. Baseline Characteristics of the Patients

Two participants eventually withdrew from the trial because they could not tolerate intubation. The remaining 68 patients with rGERD symptoms included 35 males and 33 females with a median age of 47.15 ± 13.05 years, body mass index of 22.82 ± 3.29 kg/cm [2], and GerdQ score of 8.77 ± 2.77. In this study, the 68 patients with rGERD symptoms included 15 cases of reflux esophagitis confirmed by endoscopy, two cases of Barrett's esophagus, one case of esophageal hiatal hernia accompanied by reflux esophagitis, and 51 cases of non-erosive reflux disease diagnosed by 24-hour pH monitoring off PPI. Percentage time of pH < 4 (%) and DeMeester score of the acupuncture group and the control group were 7.90 (5.10, 13.40) vs. 8.15 (4.80, 15.60) and 21.60 (14.70, 26.40) vs. 22.30 (16.10, 29.50), respectively. The baseline characteristics of the acupuncture group and the control group were listed in Table 1, and no statistically significant difference was observed between groups.

### 3.2. Efficacy and Safety of Acupuncture Therapy

**Figure 2:**
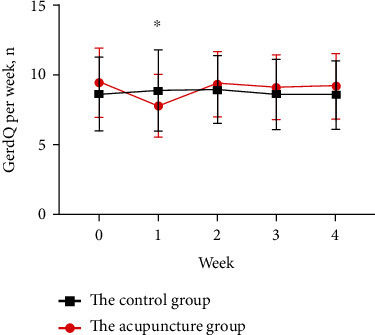
Weekly GerdQ scores during the study.

In this study, all the patients were followed up 1 week later and denied adverse reactions such as pain, hematoma, bleeding, syncope, fatigue, needle sensation, pneumothorax, organ injuries, and infections. A comparison of the changes in the GerdQ scores of patients with rGERD symptoms before and after acupuncture showed that the GerdQ scores significantly decreased in the first week after the intervention in the acupuncture group (9.45 ± 2.44 vs. 7.82 ± 2.21; *P* < 0.001) and did not change significantly in the control group (8.66 ± 2.63 vs. 8.89 ± 2.93; *P* = 0.133). However, the GerdQ scores increased to the baseline level since the second week in the follow-up period in the acupuncture group (Figure 2). Similarly, while 45.45% of patients with atypical rGERD symptoms improved in the first week after acupuncture, this change also returned to the baseline level since the second week.

### 3.3. Esophageal Body Motor Function

The mean DCI of the patients with rGERD symptoms in the acupuncture group was 1223.7 (581.65, 2827.10) mmHg s cm after acupuncture, which was higher than 1001 (531.35, 2342.10) mmHg s cm before acupuncture (*P* < 0.001). However, in the control group, the mean DCI also increased from 762.40 (541.70, 1280.40) mmHg s cm to 1198.70 (678.30, 1592.30) mmHg s cm (*P* = 0.002) (Table 2). Moreover, there was no significant difference in DL changes before and after the intervention in patients with rGERD symptoms in both groups (*P* > 0.05) (Table 2).

Individual swallowing in the acupuncture group and control group was analyzed according to Chicago classification version 3.0. Since every patient was asked to take 5 mL of water 10 times, there were 330 swallows in the acupuncture group and 350 swallows in the control group. Comparison of the contraction vigor before and after the intervention showed that the percentage of normal contraction, which was the primary study outcome, of patients with rGERD symptoms in the acupuncture group increased after acupuncture (84.85% vs. 93.33%, *P* = 0.06), while the percentage of ineffective contraction significantly decreased (13.03% vs. 5.45%, *P* = 0.001). The percentages of normal contraction (89.43% vs. 92.86%) and ineffective contraction (10.29% vs. 6.86%) in the control group showed no significant changes after intervention (Table 3).

Comparison of contraction patterns before and after the intervention showed that the percentage of intact contraction, which was the primary study outcome, in the acupuncture group significantly increased after acupuncture (87.58% vs. 96.36%, *P* = 0.023). However, the percentage of fragmented contraction in the acupuncture group significantly decreased after acupuncture (10.91% vs. 3.64%, *P* < 0.001). No significant changes were observed in the percentages of intact contraction and fragmented contraction after intervention in the control group (Table 3).

### 3.4. Barrier Function of the Esophageal Sphincter (the Secondary Study Outcome)

In the acupuncture group, the lengths of LES before and after acupuncture were 3.10 ± 1.08 cm and 3.78 ± 1.01 cm, and the difference was significant (*P* < 0.001). Comparison of the length changes of the LES before and after treatment in each patient indicated that 22 patients showed an increase in the length of LES, 4 patients did not show any change in the length of LES, and the LES length decreased in the other 7 patients. No significant change was observed in the length of LES before and after the intervention in the control group (*P* = 0.109) (Table 2).

The intra-abdominal LES length in the acupuncture group significantly increased after the intervention (2.14 ± 1.05 cm vs. 2.75 ± 1.16 cm, *P* = 0.002). In the control group, there was no significant change of intra-abdominal LES length before and after the intervention (*P* = 0.202) (Table 2).

Mean basal LES pressure increased from 22.02 ± 10.03 mmHg to 25.06 ± 11.48 mmHg after acupuncture in the acupuncture group (*P* = 0.014), and the basal LES pressure decreased in 13 patients but increased in 20 patients. The change observed in the control group before and after intervention (23.71 ± 9.92 mmHg vs. 22.63 ± 9.21 mmHg, *P* > 0.05) was not significant (Table 2). However, IRP was 11.06 ± 6.86 mmHg and 12.38 ± 9.33 mmHg before and after the intervention in the acupuncture group (*P* = 0.114), which showed no significant difference, and was the same as that in the control group (*P* = 0.412).

Mean basal UES pressure decreased significantly from 83.72 ± 106.78 mmHg to 65.91 ± 91.84 mmHg after acupuncture in the acupuncture group (*P* = 0.013), while in the control group, it decreased from 71.74 ± 42.24 mmHg to 44.89 ± 18.62 mmHg (*P* < 0.001). Moreover, there was no significant change in UES relaxation pressure in both groups (*P* = 0.986, 0.577).

## 4. Discussion

GERD is a type of esophageal and gastric motility disease caused by a variety of factors. Generally speaking, the reflux mechanism is the decline of anti-reflux defense function and the enhancement of counter-flow attack factor. GERD symptoms have multiple potential determinants, including the number of reflux episodes, the proximal extent to which the refluxate migrates, the acidity of the refluxate, esophageal hypersensitivity, and cognitive hypervigilance [12]. However, alterations of esophageal function are closely related to gastroesophageal reflux, which usually occurs via four mechanisms: transient lower esophageal sphincter relaxations (TLESRs), low LES pressure, swallow-associated LES relaxations, and straining during periods with low LES pressure [13].

Acupuncture, an important part of traditional Chinese medicine, has been used for thousands of years to treat different diseases by stimulating different combinations of acupoints. Recent studies have shown that acupuncture can improve the symptoms of GERD [6]. The effects of acupuncture on gastrointestinal motility were fairly consistent, and the major acupuncture points used in these studies were ST36 and PC6. Acupuncture at the lower limbs (ST36) causes muscle contractions via the somatoparasympathetic pathway, while acupuncture at the wrist (PC6) showed antiemetic and antinociceptive effects and may be beneficial for patients with visceral hypersensitivity [14]. Acupuncture at the margin of foot (SP4) is mainly used for vomiting, abdominal pain, diarrhea, and other gastrointestinal diseases [15]. In this study, we originally chose the acupoint combination of ST36, PC6, and SP4 to treat rGERD symptoms according to the Meridian Theory and took advantage of HRM to evaluate the instant changes in esophageal motility in patients with rGERD symptoms during the course of acupuncture.

In our study, the patients with rGERD symptoms showed significant improvements in the first week after acupuncture. Moreover, the mean basal LES pressure increased after acupuncture in 60.6% (20/33) of the patients with rGERD symptoms, which was accompanied by a significant increase in LES length and intra-abdominal LES length. Further individual swallow analysis showed that after acupuncture, the percentages of intact and normal contractions increased while the numbers of ineffective swallows and fragmented contractions significantly decreased. These results indicated that esophageal motility improved after acupuncture, consistent with the results of previous studies [16]. Abnormal esophageal motility is closely related to reflux burden. A hypotensive basal LES pressure and abdominal LES length of <1 cm have been shown to be associated with increased distal esophageal acid exposure [17–19]. Esophageal body contraction vigor measured by the DCI can predict effective peristaltic clearance of the esophagus. Failed esophageal body peristalsis may predict more severe clearance disorder and is also associated with abnormal acid exposure [20, 21], and this compounds with a low esophagogastric junction contractile integral (EGJ-CI), which is a novel HRM metric to measure the contractile vigor of the EGJ, promotes a high acid burden [22]. Therefore, one of the mechanisms by which acupuncture improves the rGERD symptoms may be to reduce abnormal esophageal acid exposure by improving esophageal motility.

However, not all patients with abnormal esophageal motility have rGERD symptoms. Esophageal hypersensitivity and cognitive hypervigilance also play important roles in the development of GERD symptoms. Another mechanism of acupuncture improving the rGERD symptoms is more likely to be related to its possible effect on visceral hypersensitivity. In patients with chest pain, acupuncture has been reported to reduce esophageal pain perception to intra-esophageal balloon distention [23]. Thus, acupuncture may achieve its therapeutic effect by modulating visceral sensation in patients with rGERD symptoms.

There are other possible mechanisms by which acupuncture improves rGERD symptoms. Previous studies have shown that acupuncture can influence the intact preparation of vagal nerves and sympathetic nerves, resulting in changes in intragastric pressure and waves of gastric contraction in rats [24]. In addition, many studies have demonstrated that acupuncture can suppress gastric acid secretion, which was mediated by the interaction of humoral and neural pathways [25]. Alternatively, acupuncture may reduce the duodeno-gastro-esophageal reflux or weakly acidic reflux by humoral and neural pathways [19].

The GerdQ scores of patients with rGERD symptoms in our study decreased in the first week after acupuncture treatment, but they rose back to the baseline level from the second to the fourth week during the follow-up period. Thus, acupuncture showed a short-term efficacy in the treatment of rGERD symptoms. Repeated acupuncture treatment at a certain interval may be needed to maintain the therapeutic effect.

Our study had some limitations: (1) the sample size of this study was relatively small; (2) the long-term efficacy of acupuncture has not been proven in patients with rGERD symptoms; (3) the patients in our study did not receive pH monitoring again after PPIs failure, so we could not confirm that the refractory symptoms were caused by insufficient acid suppression or other reasons such as esophageal hypersensitivity.

In future studies, we will investigate whether long-term effects can be achieved by increasing the frequency of acupuncture and explore the mechanisms underlying the therapeutic effects of acupuncture for rGERD symptoms.

## Figures and Tables

**Table 1:** Baseline characteristics of the acupuncture group and the control group.

Characteristic	Acupuncture group (*n* = 33)	Control group (*n* = 35)	*P*-value
Age (years)-mean (SD)	47.85 ± 13.28	45.23 ± 12.56	0.670
Gender (*N*/%)			
Male	16 (48.48%)	19 (54.28%)	0.632
Female	17 (51.52%)	16 (45.71%)	0.632
Body mass index (mean/SD)	22.90 ± 2.80	22.49 ± 3.84	0.846
Risk factors (*N*/%)			
Hypertension	3 (33.33%)	7 (20.00%)	0.204
Diabetes	1 (3.03%)	0 (0.00%)	0.299
Hyperlipidaemia	2 (6.06%)	3 (8.57%)	0.692
Cigarette smoking	4 (12.12%)	5 (14.28%)	0.792
Alcohol consumption	2 (6.06%)	2 (5.71%)	0.952
Typical symptoms (*N*/%)			
Acid reflux	15 (45.45%)	22 (62.86%)	0.150
Heartburn	14 (42.42%)	14 (40.00%)	0.839
Accompanied atypical symptoms (*N*/%)			
Pharyngeal discomfort	19 (57.57%)	16 (45.71%)	0.328
Chest pain	9 (27.27%)	12 (34.29%)	0.532
Retrosternal discomfort	12 (36.36%)	7 (20.00%)	0.133
GERD Q (mean/SD)	9.45 ± 2.44	8.66 ± 2.63	0.076
Endoscopic findings (*N*/%)			
RE	7 (21.21%)	8 (22.86%)	0.870
BE	0 (0.00%)	2 (5.71%)	0.157
Hiatus hernia	1 (3.03%)	0 (0.0%)	0.299
24-hour pH monitoring			
Percentage time pH < 4 (%) (median/IQR)	7.90 (5.10, 13.40)	8.15 (4.80, 15.60)	0.438
DeMeester score (median/IQR)	21.60 (14.70, 26.40)	22.30 (16.10, 29.50)	0.585

**Table 2:** Changes of esophageal motility after intervention between the acupuncture group and the control group.

	Acupuncture group (*n* = 33)			Control group (*n* = 35)		
	Before	After	*P*-value	Before	After	*P*-value
LES length (cm)	3.10 ± 1.08	3.78 ± 1.01	≤0.01∗	3.45 ± 0.90	3.63 ± 0.87	0.109
Intra-abdominal LES length (cm)	2.14 ± 1.05	2.75 ± 1.16	0.002∗	2.27 ± 1.01	2.35 ± 1.33	0.202
LESP (mmHg)	22.02 ± 10.03	25.06 ± 11.48	0.014∗	23.71 ± 9.92	22.63 ± 9.21	0.393
IRP (mmHg)	11.06 ± 6.86	12.38 ± 9.33	0.114	10.94 ± 4.59	11.45 ± 4.79	0.412
DCI (mmHg cm s)	1001.00 (531.35, 2342.10)	1223.70 (581.65, 2827.10)	≤0.01∗	762.40 (541.70, 1280.40)	1198.70 (678.30, 1592.30)	0.002∗
IBP (mmHg)	2.29 ± 3.20	1.22 ± 5.03	0.322	3.33 ± 4.41	0.90 ± 6.26	0.051
DL (s)	6.70 (5.90, 7.55)	6.40 (6.10, 7.85)	0.129	7.10 (6.10, 7.85)	6.88 (5.90, 7.55)	0.324
UESP (mmHg)	83.72 ± 106.78	65.91 ± 91.84	0.013∗	71.74 ± 42.24	44.89 ± 18.62	≤0.01∗
UES relaxation pressure (mmHg)	5.95 ± 2.08	6.80 ± 2.94	0.986	5.75 ± 1.49	6.03 ± 2.29	0.577

∗*p* < 0.05. LES: lower esophageal sphincter; LESP: lower esophageal sphincter pressure; IRP: integrated relaxation pressure; DCI: distal contractile integral; IBP: intrabolus pressure; DL: distal latency; UES: upper esophageal sphincter; UESP: upper esophageal sphincter pressure.

**Table 3:** Esophageal pressure topography scoring of individual swallows in the acupuncture group and the control group.

		Acupuncture group (swallow = 330)			Control group (swallow = 350)		
		Before	After	*P*-value	Before	After	*P*-value
Contraction vigor	Ineffective	43	18	0.001∗	36	24	0.104
	Normal	280	308	0.06	313	325	0.231
	Hypercontractile	7	4	0.362	1	1	1.000
Contraction pattern	Premature	5	0	0.025∗	6	4	0.524
	Fragmented	36	12	<0.001∗	37	31	0.247
	Intact	289	318	0.023∗	307	315	0.198

∗*p* < 0.05.

## Data Availability

The data used to support the findings of this study are available from the corresponding author (Yao Weiyan: ywy11419@rjh.com.cn) upon reasonable request.
